# Review of Prof. F. G. Lemercier's Lecture on Clastic Anatomical Models

**Published:** 1869-05

**Authors:** Henry R. Noel


### AMERICAN JOURNAL

OF

### DENTAL SCIENCE.

Vol. III. THIRD SERIES--MAY, 1869. No. 1.

### ARTICLE I.

### *Review of a Lecture upon Clastic Anatomical Models,*

Delivered before the Baltimore College of Dental Surgery, by F. G. Lemercier, Co-operator of Dr. Auzoux and Professor of the Polytechnic Association of Paris.

While delivering a course of lectures before Peabody Institute of Baltimore, Dr. Lemercier was engaged by the Faculty of the Baltimore College of Dental Surgery, to deliver a lecture upon "Clastic Models," and to exhibit before the class the specimens he had brought from Paris.

This Lecture was delivered March 2nd 1869, and before a large and appreciative audience, embracing the class, many physicians and dentists of the city, and the Faculty of the College.

The subject was one of the deepest interest, to the Faculty and to the class, as offering the only solution of "that vexed question," "*anatomical study by disssection.*" That a student can find time, in two or even three courses, to perfect himself in practical anatomy by dissection, is simply an utter impossibility; and to meet and obviate this great *error* in our schools has been the aim of the Faculty for some years. Anatomical study requires either the subject, or a series of finished models, the first being impracticable, the latter must be adopted from necessity. It is worse than useless to discuss the value of dissection, we all admit its value, if thorough, and yet two-thirds of the medical graduates even, are most lamentably deficient in their anatomy. Turn it over as we may, shirk it as we please—yet the patent fact stares us in the face, that dissection, as a means of acquiring a practical knowledge of anatomy, is as at present conducted in our schools, both medical and dental, an unmitigated humbug.

*It is practically a grand failure in America.*

Drs. Lemercier and Auzoux have met this great question fairly and squarely—they have prepared one of the most comprehensive series of models ever offered the profession.

The human body as a whole; then resolved into systems of organs—then into special organs; then into tissues; and finally into the minutest *naked eye* anatomical forms. Then by comparative anatomy, from the simplest rudimentary form of organ and rudimentary function, up to the most elaborately complex anatomical structure and gradual differentiation and development of the highest physiological office. There is nothing wanting even in the comparative anatomy, for the illustrating models of each organ show its earliest and simplest, as well as its final and most complex anatomy.

These models have an anatomical accuracy which commands the highest admiration, and in many instances the greatly magnified proportions give the microscopical anatomy, to the unassisted eye, with a truthfulness which leaves us mute.

"From the first appearance of the ovule in the ovary to the formation of the embryo, and about to the 30th day of pregnancy," there are more than 20 magnified models, illustrating the process in a way no dissection could possibly do. These models of the Fœtus and Uterus during each month of gestation; models illustrating the infant's 1st year and then up to adult life; and in each and every model where ever organs are distinctly marked, they are arranged "clastically," *i.e.* so arranged, that they can be removed one by one from the body, and each organ studied by itself. Not only is this true, but each organ, if of any size, is always in many pieces, showing its exact anatomical structure.

The human being from ovule to adult life, is illustrated; the system of organs and the special organs are also illustrated; and by the models from comparative anatomy the tissues and organs are traced up to their complete perfection in man; enabling us in a few months to grasp, comprehend and retain those grand truths of comparative developement, the evolution which cost Cuvier, Owens, Cruvielhier, &c., so many years of unremitting labor. To such a degree has this process of copying nature by clastic models been carried, that vegetables, fruits and flowers have all been laid under contribution and made to contribute their quota. The study of Botany is thus rendered both interesting and instructive, to those who never have seen the flowers of the country, and who depend alone upon the models for their information.

Being fully cognizant of the above facts, and appreciating the perfect failure of dissection, as at present conducted in the different schools of America, the Faculty of the Baltimore College of Dental Surgery had procured specimens and models, and among the number was " a life size clastic preparation of man " from the firm of Auzoux and Lemercier, of Paris.

This model had been used in the anatomical and physiological lectures of the session, and had given the most complete satisfaction to both students and professors, and it was with the most lively interest, and an earnest appreciation of the subject, that the class and faculty listened to the lecture.

The lecture was a description of the models, many of which were exhibited as specimens, and their anatomical relations accurately described.

Dr. Lemercier exhibited several greatly magnified specimens;—one of the hand, another of the eye, another of the ear, were probably the best and most striking.

He took up this enlarged model of the hand, and dissected it piece by piece, giving with perfect accuracy the anatomy down to the veriest minutiæ of fascia and ligament; he removed the *skin from the thumb*; showed the matrix and bed of the nail in the most beautifully correct and scientific manner. He then continued his dissection, demonstrating as he proceeded, just as the ordinary demonstrations of Anatomy are given; step by step he removed fascia, tendons, superficial muscles, arteries, veins, nerves, &c.; then the deeper muscles of thenar and hypo-thenar eminences; deeper arteries, veins, nerves, tendons; finally the interossei muscles and their attachments and the joints with their respective ligaments. Every portion was removed and the plainest skeleton of the hand alone remained; this was the anatomical analysis of the hand, then the separate constituents were one by one replaced and the hand by this synthetic action perfectly restored to its first appearance. We doubt extremely, whether one month's unbroken dissection of the hand could convey as clear an idea of its anatomy, as did that 15 minutes of analytic and synthetic study by the model.

The "Magnified Eye" was next taken up, and the severest critic could have found little indeed for censure or criticism, as he watched the incomparable manner in which the lecturer dissected and demonstrated this most complex organ. The sclerotic coat was removed first; then choroid and cornea; next the *iris* and ciliary ligament with the aqueous humor; then the crystalline lens and vitreous, with "central artery."

The retina was now brought fully into view and its anatomical structure in all its microscopical complexity carefully stated; the optic nerve, optic disc, yellow spot of Soemmering, and the radiating fibres of the optic nerve were all carefully shown.

The lecturer then gave an admirable account of the "optical mechanism of the eye" and "the optical action" of this mechanism, then of the nerve mechanism and its action; and finally explained all the movements of the eye ball by the recti and oblique muscles. He showed the distribution of the third pair of nerves to the superior, inferior and internal recti, and to the "levator palpebrae" and inferior oblique muscles; he showed the distribution of the fourth pair to the superior oblique and sixth pair to the external rectus. The distribution of the opthalmic division of the fifth pair was also beautifully exemplified.

The "Magnified Ear" was next exhibited and as thoroughly dissected, and minutely described as had been the eye; the external ear—auditory canal—"membrana tympani;" and tympanum with the chain of little bones, *malleus*, *incus* and *stapes*; and the stapadius, tensor and laxator tympani muscles; the fenestra ovalis and fenestra rotunda, Eustachian tube and mastoid cells were fully shown; the internal ear or labryrinth with its vestibule, semicircular canals and cochlea was shown, and minute descriptions given of canals and cochlea.

The anatomical details were perfect, and the portions demonstrated were one by one removed, examined, function given and then replaced.

At this stage of the lecture, all present felt the great value of these magnified models, for the internal ear is not a thing that one student in five-hundred ever dissects or ever understands, and yet there before the audience was every part laid that could belong to the normal ear;—and piece by piece they had witnessed the dissection without one drop of blood obscuring the clearness of the anatomical forms until the ear as a whole had been resolved into its constituent elements.

It is impossible in our short review, to notice all of the models exhibited, and we therefore pass to the last and most exquisite of them all.

" The models showing the nervous system from Comparative Anatomy."

To the comparative anatomists and to the comparative physiologists, this was indeed a treat rarely enjoyed, in this country. The models began with the Class *Radiata*, of which the star-fish is the type; where we have series of ganglia arranged in a more or less circular form and connected with each other by nervous cords. Then the Hetero-gangliate type, "with its ganglia and nerves around the gullet and nerves radiating symetrically to be connected with other irregular scattered ganglia;" the oyster and snails are examples:

Then the articulata-homogangliate type, "having a double ventral cord, with ganglia at short intervals,, generally a pair (right and left) coalesced on the middle line for *each* segment of the body, and communicating anteriorly by nerves surrounding the gullet, with a pair of supra œsophageal ganglia. To this class belong "earth" worms, insects &c. Finally the vertebrate class was represented, and models showing the comparative anatomy of the ganglia of special sense exhibited.

The Sensory ganglia exhibited in the order of normal developement, from Fish, Reptiles, Birds, Mammals, and up to the highest grades in the mammals. Then the cerebral ganglia were given by the same order, and the size and complexity of the convolutions were shown to coincide with the intelligence of the animal. The size of the brain and complexity of convolutions were illustrated by several models, beginning with some of the lowest species of the Monkey, and running up to the adult brain of the Caucasian. The brain of an idiot was shown, and its small size contrasted with the normal adult brain; the arrested anatomy causing arrested physiology; for with arrest of the developement of the Cerebral Ganglia, or superficies of the brain, there was a paripassu arrest of intelligence.

The Ganglia of the lowest and of the highest grades of animal life ever compared and contrasted, the functions being ascertained to a great extent by watching the anatomical mechanism and seeing its increase in size correspond with increased power of some special sense; or with increased accuracy and intensity of the manifestation of some special function belonging to the nervous system. From this comparative anatomy and from the comparative physiology of the nervous system, we are enabled to evolve certain grand truths in regard to nervous action and nervous phenomena.

1st. It teaches us that the nervous system is but a series of ganglia, connected and commissured by white cords called nerves; 2nd. That the internal and external peripheries are connected or commissured with these ganglia by similar cords or nerves.

3rd. That these cords or nerves are for conduction only,(even as the wires of the telegraph) and they conduct in distinct lines, isolated and with extreme velocity. 4th. That there are two kinds of nerves—or nerves discharging two distinct functions even in conduction:—the one called *afferent* or *Sensory*, begins in a sentient recipient expansion on the periphery internal and external, and terminates in the *nervous* ganglia or "nerve centres"; the other, *efferent* or *Motor*, begins in these same nerve centres and *returns* to the same pheriphery, and is then lost by blending in organic continuity with the tissues; it is therefore impossible to tell in every instance, where nerve tissue begins or where it ends, for in some instances it runs into and blends so imperceptibly, with other tissues that there is no line of demarcation. Afferent nerves begin in peripheral expansions and end in ganglionic masses—or nerve centres; efferent nerves begin in the ganglionic masses or nerves centres and end or terminate in peripheral expansions, or peripheral distribution; the function is the same, i e. conduction, only one conducts *to* and the other *from* nerve centres.

### *Function of Nerve Centres.*

Of course they *receive* impressions from the afferent;—of course they transmit or send out impressions by the efferent nerves; but they also diffuse—concentrate—store up or retain; they diffuse, for one nerve often enters a ganglion, and many nerves may leave this ganglion,hence any impressions brought in by the one is reflected and diffused through many *efferent* channels; they concentrate, for often several nerves enter but only one emerges from a ganglion, hence in this case the *many* impressions by the afferent are concentrated and sent out by *one efferent* nerve ; they retain or store up—for often the impressions brought to the nerve centre are not all reflected, but a portion is stored up or retained and after some marked interval is allowed to escape—or may even be retained for an indefinite period—in this case the ganglion is said to "*register*" the impression.

Ganglia therefore have several different modifications of nervous action ; they receive impressions ;—transmit and reflect impressions ;—diffuse and concentrate impressions ;—retain or store up impressions ; now a careful consideration of the above, will at once prove, that ganglia are capable of altering, modifying, combining and even taking off and registering, thus *for the time, destroying impressions.*

Now there is another function of the ganglia, or at least another power which the ganglia exhibits when acted upon by its appropriate agent ; it is the power of "elaboration." The power of collecting registered impressions and combining and interweaving them, so as to produce new combinations—new results. For this process to take place three elements are necessary :—1st afferent nerves to receive and conduct impressions ; 2nd ganglia to receive and register these impressions ; 3rd an agent to act upon the ganglia—to resurect as it were these registered impressions and to combine and elaborate them.

" The nerves of special sense and afferent nerves generally, collect impressions ;—the intra-cranial ganglia register these impression ;—the *soul* acting upon the brain is the agent which elaborates them in the process of "mental action," vide Draper's Human Pysiology, page 285 &c.

Now having determined the functions of nerves and of nerve centres, we will return to Dr. Lemercier and his models.

The models of the class Articulata, show a double ventral cord ;—or a cord consisting of ganglia commissured on the same horizontal line, each with its neighbor in front and its neighbor behind ; then each ganglia was connected by another short commissure with a *band of nerves*, or nervous matter of fibrous form, immediately above it ; so each ganglia had two horizontal and one vertical commissure excepting the first and last, these of course had only one horizontal commissure each; now each ganglia could be shown to consist of two ganglia, placed side by side and coalescing on the median line; the ganglia discharge the usual functions of ganglia and the cord or band above the ganglia, is a band of nerves. commissuring these ganglia with another series of ganglia, situated not beneath the belly but above it, and above the œsophagus and called ".supra œsophageal ganglia." These supra œsophageal ganglia a sort of a bilobed mass, correspond in function with the brain of the higher classes.

This bilobed mass is the seat of the instinct, and the band of nerves subventral, connects the subventral ganglia with this bilobed mass, i.e. it is a commissure. Tracing by comparative anatomy, the gradual development of this subventral cord and the subventral longitudinal series of ganglia, we are enabled to mark the blending of the two until we reach the vertebrate type, and here the *position* is also changed; for the cord is no longer subventral but dorsal, and enclosed in a jointed bony canal—in this class it is called the " spinal cord."

Now this "spinal cord" presents the appearance of a mass of whitish nerves or cords, and only when we cut into its symmetrical halves right and left do we find the greyish ganglionic substance and find it commissured on the median line.

Ganglia so distinct in the articulata, have been completely surrounded, enveloped hidden from view by the commissural band; and not only is this true, but these ganglia have been so crowded together, that they communicate right and left, and run into each other above and below, thus there is a loss of anatomical individuality in the ganglia, and a blending in organic continuity of a continuous extension of ganglionic substance. The white commissural band envelopes this extension of ganglionic matter and we have the "spinal cord."

The functions of this cord are: 1st, physico-reflex in virtue of its coalesced and blended ganglia, 2d commissural in virtue of its band of white nerves and of some grey cords also, the analogy between articulata and the vertebrata hold good in the physiology as well as the anatomy.

*To be Continued.*

---

### ARTICLE. II.

### *Microscopy of the Dental Tissues. New Series.*

By S. P. Cutler, M.D.,A.E.G.,D.D.S,

*Continued.*

